# Significance of dietary quinoa husk (*Chenopodium quinoa*) in gene regulation for stress mitigation in fish

**DOI:** 10.1038/s41598-024-58028-4

**Published:** 2024-04-01

**Authors:** Neeraj Kumar, Supriya Tukaram Thorat, Aliza Pradhan, Jagadish Rane, Kotha Sammi Reddy

**Affiliations:** ICAR-National Institute of Abiotic Stress Management, Malegaon, Baramati, Pune, 413115 India

**Keywords:** Quinoa, Abiotic stress, Caspase, *CYP450*, *iNOS*, Fish, Animal physiology, Ichthyology

## Abstract

The persistent challenges posed by pollution and climate change are significant factors disrupting ecosystems, particularly aquatic environments. Numerous contaminants found in aquatic systems, such as ammonia and metal toxicity, play a crucial role in adversely affecting aquaculture production. Against this backdrop, fish feed was developed using quinoa husk (the byproduct of quinoa) as a substitute for fish meal. Six isonitrogenous diets (30%) and isocaloric diets were formulated by replacing fish meal with quinoa husk at varying percentages: 0% quinoa (control), 15, 20, 25, 30 and 35%. An experiment was conducted to explore the potential of quinoa husk in replacing fish meal and assess its ability to mitigate ammonia and arsenic toxicity as well as high-temperature stress in *Pangasianodon hypophthalmus*. The formulated feed was also examined for gene regulation related to antioxidative status, immunity, stress proteins, growth regulation, and stress markers. The gene regulation of *sod, cat, and gpx* in the liver was notably upregulated under concurrent exposure to ammonia, arsenic, and high-temperature (NH_3_ + As + T) stress. However, quinoa husk at 25% downregulated *sod, cat*, and *gpx* expression compared to the control group. Furthermore, genes associated with stress proteins *HSP70* and *DNA* damage-inducible protein (*DDIP*) were significantly upregulated in response to stressors (NH_3_ + As + T), but quinoa husk at 25% considerably downregulated *HSP70* and *DDIP* to mitigate the impact of stressors. Growth-responsive genes such as myostatin (*MYST*) and somatostatin (*SMT*) were remarkably downregulated, whereas growth hormone receptor (*GHR1 and GHRβ*), insulin-like growth factors (*IGF1X, IGF2X*), and growth hormone gene were significantly upregulated with quinoa husk at 25%. The gene expression of apoptosis (*Caspase 3a* and *Caspase 3b*) and nitric oxide synthase (*iNOS*) were also noticeably downregulated with quinoa husk (25%) reared under stressful conditions. Immune-related gene expression, including immunoglobulin (*Ig*), toll-like receptor (*TLR*), tumor necrosis factor (*TNFα*), and interleukin (*IL*), strengthened fish immunity with quinoa husk feed. The results revealed that replacing 25% of fish meal with quinoa husk could improve the gene regulation of *P. hypophthalmus* involved in mitigating ammonia, arsenic, and high-temperature stress in fish.

## Introduction

The demand for fishmeal and fish oil has significantly increased in recent decades, driven by the continuous needs of the aquaculture industry^1^. The excessive use of fishmeal in fish feed has resulted in elevated costs and is not sustainable for long-term aquaculture production. Consequently, the quest for new, alternative, and efficient fishmeal substitutes has become increasingly urgent^2,3^. Plant proteins have been extensively studied as fishmeal substitutes in aquafeeds, including soybean meal^4–7^, rapeseed meal^8^ (Cheng et al., 2010), cottonseed meal^9^, and peanut meal^10^. However, plant proteins have challenges such as the presence of anti-nutritional factors, low feed availability, and an unbalanced amino acid profile, which negatively impacting various fish species^11^. The United Nations declared 2013 as the International Year of Quinoa in recognition of its significant potential. Interestingly, quinoa husk has emerged as a promising alternative for replacing fishmeal in fish feed. Quinoa possesses exceptional nutritional value, including a high concentration of protein, unsaturated fatty acids, gluten-free content, and essential amino acids, with a low glycemic index (GI)^12,13^. Additionally, quinoa exhibits antioxidant potential, antimicrobial properties, and anti-inflammatory activities, establishing its value as a functional food^14,15^. Phenolic and flavonoid compounds, as part of quinoa's antioxidant capacity, protect organisms against free radicals and oxidative stress, promoting overall health^16^.

The present study addresses pollution in aquaculture, specifically ammonia and arsenic toxicity, which can be exacerbated by temperature fluctuations. Elevated temperatures can intensify the toxicity of ammonia and arsenic due to changes in metal speciation and water contaminants^17,18^. Ammonia pollution originates from agricultural runoff and the decomposition of biological waste in aquatic systems, posing a significant threat to aquatic animals. Ammonia's toxicity arises as NH_4_^+^ displaces K^+^ and depolarizes neurons, leading to NMDA-type glutamate receptor activation, excessive Ca^2^ + influx, and subsequent central nervous system cell death^19,20^. Total ammonia exists in two forms, ionized (NH_4_^+^) and un-ionized (NH_3_), with NH_3_ diffusing through biological cell membranes and inducing toxicity^21^. Uneaten feed and fish excreta also contribute to ammonia toxicity in aquaculture, impairing vital organs and causing mass mortality among aquatic animals^22^.

Waterborne arsenic stands as a significant global health concern, prevalent in water and food commodities. Even low doses pose severe health risks, including cancer^23^. The toxicity of arsenic depends on its chemical form, with arsenic pentavalent (As(V)) being highly toxic and trivalent arsenic (As (III)) demonstrating therapeutic potential for autoimmune and inflammatory diseases (Kumar et al^17^). Over 200 million people in countries such as India, Bangladesh, Argentina, China, Ghana, the USA, and Vietnam face a high risk of arsenic exposure^24,25^.

Molecular approaches play a crucial role in investigating the efficacy of nutrients like quinoa husk in mitigating multiple stresses. Genes associated with oxidative stress, genotoxicity, and stress proteins are upregulated in response to ammonia, arsenic, and high-temperature stress^26^, potentially inhibiting enzymatic function, depleting cellular GSH, and promoting DNA oxidation^27^. The expressions of genes such as *SOD, CAT, GPx, HSP, iNOS, MT, DNA* damage-inducible protein, *TNFα, TLR, IL, Ig, GH, GHR1, GHRβ, MYST*, and *SMT* are highly affected by arsenic pollution, ammonia, and high-temperature stress. This investigation underscores quinoa's essential role in immunomodulation in fish against stress^28^. Arsenic bioaccumulation in various fish tissues depends on biotransformation and involvement in redox and methylation reactions^29^, potentially reducing fish immunity by suppressing cytokine and antibody production^30^.

*Pangasianodon hypophthalmus* emerges as a promising candidate fish species for evaluating different feed ingredients, known for its ability to thrive in adverse conditions and tolerate various abiotic and biotic stresses^31,32^. Global *P. hypophthalmus* production reached 2520.4 thousand tonnes in 2020^33^, making it suitable for diversifying aquaculture and increasing fish production. This study has two objectives: (1) to standardize protein replacement from fishmeal using quinoa husk and (2) to assess the potential role of quinoa husk in mitigating stress through molecular approaches in *P. hypophthalmus*.

## Materials and methods

### Ethics statement

In the present study, we adhered rigorously to the ARRIVE (Animal Research: Reporting of In Vivo Experiments) guidelines. The methodology and endpoints of the results were approved by the Director of the institute and the Research Advisory Committee (RAC) of ICAR-NIASM. Additionally, we strictly followed international and national guidelines for the care and maintenance of animals during the experiment.

### Experimental animal and design

The fish were obtained from the farm pond of ICAR-National Institute of Abiotic Stress Management. The average weight and size of the fish were 7.33 ± 0.18 g and 4.92 ± 0.28 cm, respectively. They were kept in rectangular plastic tanks with a capacity of 150 L and underwent a quarantine process involving a 1% dip salt solution and potassium permanganate (KMnO4). For this experiment, a total of 12 treatments were designed, including a control group, concurrent exposure to arsenic, ammonia, and high temperature (As + NH_3_ + T), and quinoa husk (QH) fed groups at 15%, 20%, 25%, 30%, and 35% per kilogram of diet, both with and without the stressors. The treatments were labelled as follows: (1) Control; (2) Concurrent exposure to arsenic, ammonia, and high temperature (As + NH_3_ + T); (3). Fed with quinoa husk (QH) at 15% kg^−1^ diet; (4). Fed with quinoa husk (QH) at 20% kg^−1^ diet; (5). Fed with quinoa husk (QH) at 25% kg^−1^ diet; (6). Fed with quinoa husk (QH) at 30% kg^−1^ diet; (7). Fed with quinoa husk (QH) at 35% kg^−1^ diet; (8). Fed with quinoa husk (QH) at 15% kg^−1^ diet and concurrent exposure to arsenic, ammonia, and high temperature; (9). Fed with quinoa husk (QH) at 20% kg^−1^ diet and concurrent exposure to arsenic, ammonia, and high temperature; (10). Fed with quinoa husk (QH) at 25% kg^−1^ diet and concurrent exposure to arsenic, ammonia, and high temperature; (11). Fed with quinoa husk (QH) at 30% kg^−1^ diet and concurrent exposure to arsenic, ammonia, and high temperature; (12). Fed with quinoa husk (QH) at 35% kg^−1^ diet and concurrent exposure to arsenic, ammonia, and high temperature. The treatment details are also presented in Table 1. The QH diets were administered to the fish twice daily at 9:00 AM and 5:00 PM, with faecal matter and uneaten feeds removed by siphoning daily. Water quality parameters were periodically analyzed using the APHA method^34^, and these parameters remained well within the normal range for this fish species^32^. Every alternate day, 2/3rd of the water was manually changed, and (NH_4_)_2_SO_4_ was added as a source of ammonia toxicity (NH_3_), along with arsenic (sodium arsenite, NaAsO_2_). Aeration was provided throughout the experiment via a compressed air pump. Ammonium sulfate (1/10th of LC_50_ 2.0 mg L^−1^ of (NH4)_2_SO_4_) (Kumar et al^18^.), and arsenic (1/10th of LC_50_ 2.68 mg L^−1^ of arsenic)^17^, as well as a high temperature of 34 °C, were maintained to induce stress throughout the experiment. Six quinoa husk diets with iso-caloric (356.07 kcal/100 g) and iso-nitrogenous (35% crude protein) pelleted formulations were prepared. The feed ingredients included wheat flour, groundnut meal, soybean meal, fishmeal, and quinoa husk. Cod liver oil, lecithin, vitamin C, and other labile nutrients were added after heating the feed ingredients. Proximate analysis was conducted using the AOAC^35^ method; ether extract (EE) was determined through solvent extraction, and crude protein content was determined based on nitrogen content. Ash content was determined using a muffle furnace at 550 °C. Total carbohydrate content was calculated using the formula: 100-(CP% + EE% + Ash %). Additionally, the gross energy content was determined using the Halver method^36^ (Table 2).

**Table 1:** Details of Experimental design and treatments.

S. no	Details of the treatments	Notation
1	Control	Ctr
2	Fed with control diet and concurrent exposure to arsenic, ammonia and high temperature	As + NH_3_ + T
3	Fed with quinoa husk (QH) at 15% kg^−1^ diet	QH at 15% kg^−1^ diet
4	Fed with quinoa husk (QH) at 20% kg^−1^ diet	QH at 20% kg^−1^ diet
5	Fed with quinoa husk (QH) at 25% kg^−1^ diet	QH at 25% kg^−1^ diet
6	Fed with quinoa husk (QH) at 30% kg^−1^ diet	QH at 30% kg^−1^ diet
7	Fed with quinoa husk (QH) at 35% kg^−1^ diet	QH at 35% kg^−1^ diet
8	Fed with quinoa husk (QH) at 15% kg^−1^ diet and concurrent exposure to arsenic, ammonia and high temperature and	QH at 15% kg^−1^ diet + As + NH_3_ + T
9	Fed with quinoa husk (QH) at 20% kg^−1^ diet and concurrent exposure to arsenic, ammonia and high temperature and	QH at 20% kg^−1^ diet + As + NH_3_ + T
10	Fed with quinoa husk (QH) at 25% kg^−1^ diet and concurrent exposure to arsenic, ammonia and high temperature and	QH at 25% kg^−1^ diet + As + NH_3_ + T
11	Fed with quinoa husk (QH) at 30% kg^−1^ diet and concurrent exposure to arsenic, ammonia and high temperature and	QH at 30% kg^−1^ diet + As + NH_3_ + T
12	Fed with quinoa husk (QH) at 35% kg^−1^ diet and concurrent exposure to arsenic, ammonia and high temperature and	QH at 35% kg^−1^ diet + As + NH_3_ + T

**Table 2:** Ingredient composition and proximate analysis of experimental diets (% dry matter) of quinoa husk (QH), fed to *P. hypophthalmus* during the experimental period of 105 days.

	Q-0	Q-15	Q-20	Q-25	Q-30	Q-35
Fish meal	30	25.5	24	22.5	21	19.5
Groundnut meal	10	10	10	10	10	10
Soyabean	10	10	10	10	10	10
Wheatflour	18	18	18	18	18	18
Quinoa husk	0	12.3	16.4	20.5	24.55	28.64
Veg oil	3	3	3	3	3	3
Cod liver oil	2	2	2	2	2	2
Vit-mineral mixture	2	2	2	2	2	2
Cellulose	23	15.2	12.6	10	7.45	4.86
Lecithin	2	2	2	2	2	2
Total	100	100	100	100	100	100
Proximate composition of the diets						
Crude protein (CP)	34.45 ± 0.27	35.20 ± 0.06	34.82 ± 0.02	35.21 ± 0.01	35.19 ± 0.05	34.97 ± 0.15
Ether extract (EE)	9.13 ± 0.06	9.24 ± 0.04	9.23 ± 0.04	9.27 ± 0.05	9.43 ± 0.03	9.59 ± 0.11
Total carbohydrate (TC)	39.80 ± 0.37	37.43 ± 0.38	35.90 ± 1.33	37.44 ± 0.06	37.87 ± 0.13	38.02 ± 0.17
Organic matter (OM)	90.75 ± 0.04	90.19 ± 0.49	89.61 ± 0.74	90.19 ± 0.24	90.72 ± 0.08	90.69 ± 0.03
Dry matter (DM)	60.20 ± 0.11	62.57 ± 0.18	64.10 ± 1.33	62.56 ± 0.08	62.13 ± 0.18	61.98 ± 0.27
Digestible energy (DE)	360.93 ± 1.07	355.19 ± 1.71	357.48 ± 2.74	355.47 ± 0.62	358.22 ± 2.74	359.11 ± 1.94
Saponin (gram/100 g)	0.75	0.74	0.65	0.71	0.63	0.94

^a^Procured from local market, ^b^Himedia Ltd, Himedia Ltd, ^c^SD Fine Chemicals Ltd., India.

* Manual prepared Vitamin mineral mixture; Composition of vitamin mineral mix (quantity/250 g starch powder): vitamin A 55,00,00 IU; vitamin D3 11,00,00 IU; vitamin B1:20 mg; vitamin E 75 mg; vitamin K 1,00 mg; vitamin B12 0.6 mcg; calcium pantothenate 2,50 mg; nicotinamide 1000 mg; pyridoxine: 100 mg; Zn 500 mg; I 1,00 mg; Fe 750 mg; Cu 200 mg; Co 45 mg; Ca 50 g; P 30 g; Se: 2 ppm.

Digestible energy (DE) (Kcal/100 g) = (% CP × 4) + (% EE × 9) + (TC × 4).

Data expressed as mean ± SE, n = 3.

### RNA isolation and quantification

The TRIzol method was employed for total RNA isolation from the liver tissue of *P. hypophthalmus*. Liquid nitrogen was used to homogenize the liver tissue. Subsequently, chloroform was added to the homogenized samples and incubated for 5 min to facilitate phase separation. The mixture was then centrifuged to separate the RNA, followed by a wash with 75% ethanol and air drying. The resulting RNA pellet was dissolved in distilled water and stored at − 80 °C for future use. To assess RNA integrity, a 1% agarose gel was used, and RNA bands were visualized using a Gel Documentation system (ChemiDocTM MP imaging system, Bio-Rad). For RNA quantification, a NanoDrop spectrophotometer (Thermo Scientific) was employed.

### cDNA synthesis and quantitative PCR

The RevertAid First Strand cDNA Synthesis Kit (Thermo Scientific) was employed for cDNA synthesis. To eliminate trace amounts of DNA, DNase I was applied. The reaction mixture included 15 pmol of oligo dT primers, 100 ng of RNA template, and a total volume of 12 µl. This mixture was initially heated for 5 min at 65 °C and then quickly chilled on ice. Following this, 1.0 µl of reverse transcriptase enzyme, 2 µl of dNTP Mix (10 mM), and 1 µl of Ribo Lock RNase Inhibitor (20 U/µL) were added to the chilled mixture, followed by a brief centrifugation step. The reaction mixture was then incubated for 42 min at 60 °C, followed by a final step at 70 °C for 5 min, and the synthesized cDNA was stored at -20 °C. β-actin was employed as a reference to confirm the successful synthesis of cDNA. Real-time PCR was performed using SYBR Green (Bio-Rad) in conjunction with gene-specific primers. The quantification of gene expression followed a protocol consisting of an initial denaturation step for 10 min at 95 °C, amplification of the cDNA for 39 cycles, denaturation at 95 °C for 15 s, and annealing at 60 °C for 1 min^37^. Detailed information regarding the primers mentioned in Table 3.

**Table 3:** Details of primer for relative quantitative real-time PCR.

Gene	Primer sequence (5′–3′)	Accession number
*SOD*	F-GTCCATCTTACCCGGTGCCC	XM_034299545.1
	R-CGAGAGAAGACCCGGAACGC	
*CAT*	F-AGCAGGCGGAGAAGTACCCA	XM_026919141.2
	R-GCTGCTCCACCTCAGCGAAA	
*GPx*	F-GTCACTGCAGGATGCAACAC	XM_026947312.2
	R-TTGGAATTCCGCTCATTGAT	
*HSP 70*	F-CTCCTCCTAAACCCCGAGTC	XM_026934573.2
	R-CCACCAGCACGTTAAACACA	
*iNOS*	F-ACACCACGGAGTGTGTTCGT	XM_026931613.2
	R-GGATGCATGGGACGTTGCTG	
DNA damage inducible protein	F-CACCTTCGCCCTCGAAGTCT	XM_026938137.2
	R-GCTCGGGTGAGGTCTCTCAG	
*TNFα*	F-TGGAGTTCTGCTTGCCGTGG	XM_026942329.2
	R-GCAGCCTTTGCAGTCTCGGA	
*TLR*	F: TCACCACGAACGAGACTTCATCC	XM_026916808.2
	R : GACAGCACGAAGACACAGCATC	
*Ghr1*	FTATTGGCTACAGCTCGCCGC	XM_034306157.1
	R-AATCACCCCGACTGTGCTGC	
*Ghrb*	F-TTGAGCTTTGGGACTCGGAC	XM_026942987.2
	R-CGTCGATCTTCTCGGTGAGG	
*IGF-1X1*	F-GCAACGGCACACAGACACGC	XM_034313382.2
	R-CAGACGTTCCCTCACCATCCTCT	
*IGF-1X2*	F-CGAGAGCAACGGCACACAGA	XM_034313383.2
	R-TTCTGATGGACCTCCTTACAAGATG	
*IL*	F-AGCAGGATCCATCAAAGTGG	XM_026918084.2
	R-GTGCTCCAGCTCTCTGGGTA	
*Ig*	F-GGCCAGTAATCGTACCTCCA	XM_026923540.2
	R-CTTCGTAAGGTCCCCACTGA	
*MYST*	F-GGGAAAGACCTGGCCGTGAC	XM_026910492.2
	R-TCGAGGCCGGATTCTCGTCT	
*SMT*	F-CTCTGGGTGGCAGAATGAAT	XM_026921272.2
	R-AACATGAAGAGAACGTTTTCCAG	
*GH*	F-CCCAGCAAGAACCTCGGCAA	GQ859589.1
	R-GCGGAGCCAGAGAGTCGTTC	
*CYP P450*	F-GATTCGGCATCCGTGCGTGC	NC_047599.1
	R-GATGTGGCTGGGACGAGCA	
*MT*	F-CACGGCTTTTCCTGTCCGCT	AF087935.1
	R-AACAGCGCCCCCAGGTGTC	
*Cas 3a*	F-CGGCATGAACCAGCGCAAC	NC_047622.1
	R-TCCACCGCACCATCTGTCCC	
*Cas3b*	F-AGCTTTCCGTGAGCTGGGCT	NC_047601.1
	R-TGGCTGACTTGCTGTGGTCCT	
Na^+^K^+^*ATPase*	F-AACTACAAGCCCACGTACCA	XM_026923907.3
	R-CTTGCCAGCCTTAAAGCCAA	
*β-Actin*	F-CAGCAAGCAGGAGTACGATG	XM_031749543.1
	R-TGTGTGGTGTGTGGTTGTTTTG	

*SOD*: Superoxide dismutase; *CAT*: Catalase; *GPx*: Glutathione peroxidase; *HSP*: Heat shock protein; *iNOS*: Nitric oxide synthase; *TNFα*: Tumor necrosis factor; *TLR*: Toll like receptor; *Ghr*: Growth hormone receptor; *IL*; Interleukin; Ig: Immunoglobulin; *MYST*: myostatin *SMT*; Somatostatin; *CYP P450*: Cytochrome P450; *MT*: Metallothionine; *Cas 3a* and *3b*: caspase 3; *GH*: Growth hormone; *IGF1* and 2: Insulin like growth factor.

### Genes

The genes were investigated in liver tissues in this study viz. catalase (*CAT*), glutathione-s-transferase (*GST*), superoxide dismutase (*SOD*), nitric oxide synthase (*iNOS*), heat shock protein (*HSP 70*), Caspase 3a (*CAS 3a* and *3b*), cytochrome P450 (*CYP 450*), tumor necrosis factor (*TNFα*), toll like receptor (*TLR*), metallothionine (*MT*), growth hormone receptor (*Ghr1* and *Ghrb*), interleukin (*IL*), immunoglobulin (*Ig*), growth factor 1 and 2 (*IGF1* and *IGF 2*)somatostatin (*SMT*), myostatin (*MYST*), insulin like and growth hormone (*GH*), studied for real-time quantification.

### Cortisol

ELISA kit was used for cortisol determination (Catalog no. 500360, Cayman Chemicals, USA) and followed the protocol provided by the kit. The final reading was obtained using ELISA plate reader (Biotek India Pvt. Ltd.).

### Growth performance

The growth performance was determined by evaluating following method. The sampling/weighing of the fish was observed by every 15 days up to 105 days.

$${\text{FCR }} = \, {{{\text{Total}}\,{\text{dry}}\,{\text{feed}}\,{\text{intake}}\,\left( {\text{g}} \right)} \mathord{\left/ {\vphantom {{{\text{Total}}\,{\text{dry}}\,{\text{feed}}\,{\text{intake}}\,\left( {\text{g}} \right)} {{\text{Wet}}\,{\text{weight}}\,{\text{gain}}\,\left( {\text{g}} \right)}}} \right. \kern-0pt} {{\text{Wet}}\,{\text{weight}}\,{\text{gain}}\,\left( {\text{g}} \right)}}$$

$${\text{SGR}}\, = \,{1}00 \, {{\left( {{\text{ln}}\,{\text{FBW}} - {\text{ln}}\,{\text{IBW}}} \right)} \mathord{\left/ {\vphantom {{\left( {{\text{ln}}\,{\text{FBW}} - {\text{ln}}\,{\text{IBW}}} \right)} {{\text{number}}\,{\text{of}}\,{\text{days}}}}} \right. \kern-0pt} {{\text{number}}\,{\text{of}}\,{\text{days}}}}$$

$${\text{Weight gain }}\left( \% \right)\, = \,{{{\text{Final}}\,{\text{body}}\,{\text{weight}}\,\left( {{\text{FBW}}} \right) - {\text{Initial}}\,{\text{body}}\,{\text{weight}}\,\left( {{\text{IBW}}} \right)} \mathord{\left/ {\vphantom {{{\text{Final}}\,{\text{body}}\,{\text{weight}}\,\left( {{\text{FBW}}} \right) - {\text{Initial}}\,{\text{body}}\,{\text{weight}}\,\left( {{\text{IBW}}} \right)} {{\text{Initial}}\,{\text{body}}\,{\text{weight}}\,\left( {{\text{IBW}}} \right)\, \times \,{1}00}}} \right. \kern-0pt} {{\text{Initial}}\,{\text{body}}\,{\text{weight}}\,\left( {{\text{IBW}}} \right)\, \times \,{1}00}}$$

$${\text{Relative}}\,{\text{feed}}\,{\text{intake}},\,\left( {{\text{FI}}} \right)\,\left( {\% /{\text{d}}} \right)\, = \,{1}00\, \times \,({{{\text{TFI}}} \mathord{\left/ {\vphantom {{{\text{TFI}}} {{\rm I}{\text{BW}}}}} \right. \kern-0pt} {{\rm I}{\text{BW}}}})$$

$${\text{PER = }}{{{\text{Total}}\,{\text{wet}}\,{\text{weight}}\,{\text{gain}}\,{\text{(g)}}} \mathord{\left/ {\vphantom {{{\text{Total}}\,{\text{wet}}\,{\text{weight}}\,{\text{gain}}\,{\text{(g)}}} {{\text{crude}}\,{\text{protein}}\,{\text{intake}}\,{\text{(g)}}}}} \right. \kern-0pt} {{\text{crude}}\,{\text{protein}}\,{\text{intake}}\,{\text{(g)}}}}$$

$${\text{Thermal}}\,{\text{growth}}\,{\text{coefficient}},\,\left( {{\text{TGC}}} \right)\, = \,({\text{FBW}}^{{{1 \mathord{\left/ {\vphantom {1 3}} \right. \kern-0pt} 3}}} {-}{\text{IBW}}^{{{1 \mathord{\left/ {\vphantom {1 3}} \right. \kern-0pt} 3}}} )\, \times \,\left( {\Sigma {\text{D}}0} \right)^{{ - {1}}}$$

where ΣD0 is the thermal sum (feeding days × average temperature, °C)

$${\text{Daily}}\,{\text{growth}}\,{\text{index}},\,{\text{DGI}}\,\left( \% \right)\, = \,({\text{FBW}}^{{{1}/{3}}} {-}{\text{IBW}}^{{{1}/{3}}} )/{\text{days}}\, \times \,{1}00.$$

### Arsenic analysis in fish tissues and experimental water

Liver, muscle, gill, brain, and kidney tissues were collected to determine arsenic concentrations. These experimental water samples and tissues were processed using a microwave digestion system (Microwave Reaction System, Multiwave PRO, Anton Paar GmbH, Austria, Europe) and analyzed using Inductively Coupled Plasma Mass Spectrometry (ICP-MS) (Agilent 7700 series, Agilent Technologies, USA), following the method described by Kumar et al.^38,39^.

### Statistics

The data were analysed using Statistical Package for the Social Sciences (SPSS) version 16 software. Normality and homogeneity of variance were assessed using the Shapiro–Wilk and Levene's tests, respectively. One-way ANOVA (Analysis of Variance) followed by Duncan’s multiple range tests were applied in the present study. The significance level for data analysis was set at *p* < 0.05.

### Consent to participate

All authors are aware and agree with this submission for publication.

## Results

### Primary stress response

**Figure 1:**
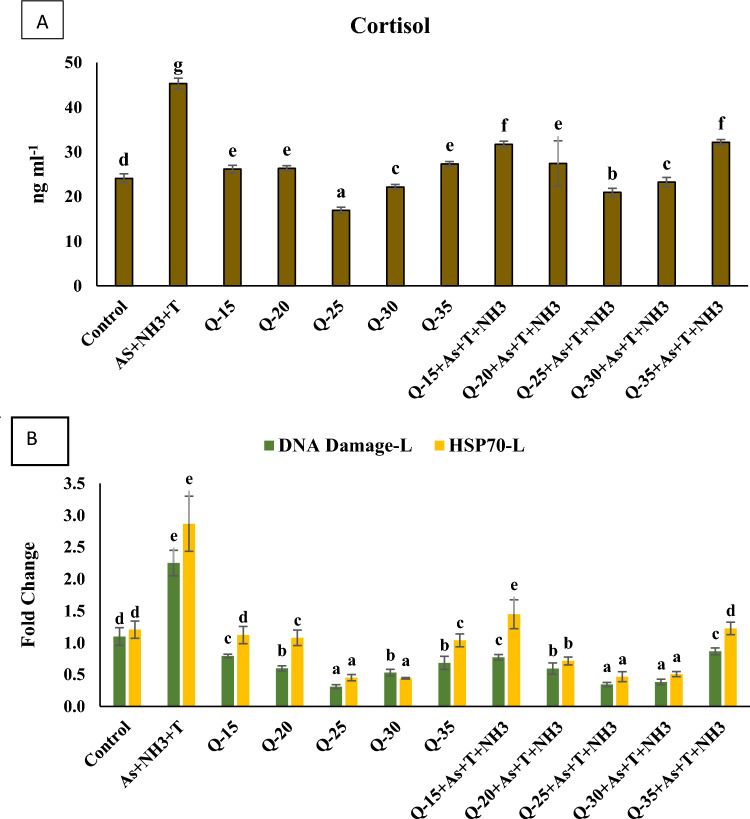
(**A**–**B**): Effect of quinoa husk on cortisol, and DNA damage inducible protein (*DDIP*) as well as heat shock protein (*HSP-70*) in fish liver. Within endpoints and groups, bars with different superscripts differ significantly (**a**–**g**). Data expressed as Mean ± SE (n = 3).

In the present investigation, fishmeal was substituted with quinoa husk (QH) at levels of 15%, 20%, 25%, 30%, and 35% in the fish feed. The objective of this study was to evaluate gene expression related to primary, secondary, and tertiary stress responses. The primary stress response, as indicated by cortisol levels, was examined in this study, and the data are presented in Fig. 1A. Cortisol levels were significantly elevated (*p* = 0.0017) in the group exposed to concurrent arsenic, ammonia, and high-temperature (34 °C) compared to the control group and those fed with QH diets, with and without stressors. Notably, the group fed with QH at 25%, with or without stressors, exhibited a significant reduction in cortisol levels compared to the control group and other supplemented groups. This study suggests that replacing fishmeal with QH at a 25% level yielded the most promising results in reducing cortisol levels in fish.

### Impact of quinoa husk (QH) on gene regulation involved secondary stress response

#### DNA damage inducible protein (*DDIP*) and heat shock protein (*HSP 70*)

The gene regulation of *DDIP* and *HSP70* in liver tissue was evaluated in fish exposed to multiple stress conditions and fed with varying levels of QH diets. The data are presented in Fig. 1B. *DDIP* (*p* = 0.001) and *HSP70* (*p* = 0.001) gene expressions were significantly upregulated in response to concurrent exposure to ammonia, arsenic, and high-temperature stress compared to the control group and other experimental groups. Furthermore, the groups fed with QH diets at 25% and 30%, with or without exposure to stressors (As + NH_3_ + T), exhibited a significant downregulation of *DDIP* and *HSP70* gene expressions compared to the control group and other experimental groups. It was observed that replacing fishmeal with QH at levels of 15%, 20%, and 35% was less effective in regulating the gene expression of *DDIP* and *HSP70*.

#### Apoptosis gene regulation (*Caspase 3a *and *3b*)

**Figure 2:**
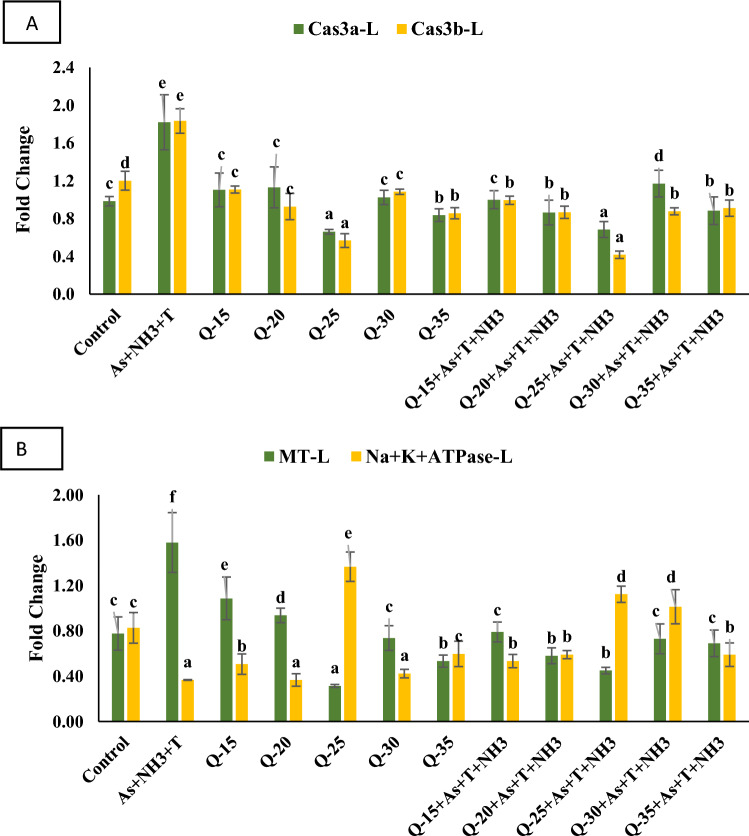
(**A**–**B**): Effect of quinoa husk on *caspase 3* and *3b*, metallothionein (*MT*) and Na^+^K^+^*ATPase* in fish liver. Within endpoints and groups, bars with different superscripts differ significantly (**a**–**g**). Data expressed as Mean ± SE (n = 3).

The impact of replacing fishmeal with QH at levels of 15%, 20%, 25%, 30%, and 35% on apoptosis genes *Cas 3a* and *3b* was assessed, and the data are presented in Fig. 2A. Apoptosis genes *Cas 3a* (*p* = 0.002) and *3b* (*p* = 0.001) showed significant upregulation in group exposed to concurrent ammonia, arsenic, and high-temperature stress, as well as fed with the control diet, in comparison to the control group and the QH-fed diet group. However, *Cas 3a* and *3b* were notably downregulated in the group fed with a 25% QH diet, both with and without stressors, in comparison to the control group and the other experimental groups.

#### Metallothionine (***MT***) and ***Na***^+^***K***^+^***ATPase***

The gene regulation of metallothionein (*MT*) and *Na*^+^*K*^+^*ATPase* was assessed in the liver tissue of *P. hypophthalmus*. The concurrent exposure to ammonia, arsenic, and high-temperature stress significantly upregulated (*p* = 0.0027) the gene regulation of *MT* in the liver tissue. Conversely, the group fed with a 25% QH diet, with or without stressors (As + NH_3_ + T), notably downregulated *MT* compared to the control group and other experimental groups (Fig. 2B). Additionally, the gene regulation of *Na*^+^*K*^+^*ATPase* was significantly upregulated (*p* = 0.0012) by the 25% QH diet, while it was downregulated by concurrent exposure to ammonia, arsenic, and high-temperature stress, in comparison to the control group and other experimental groups (Fig. 2B).

#### Inducible nitric oxide synthase (*iNOS*) and Cytochrome P450 (*CYP 450*)

**Figure 3:**
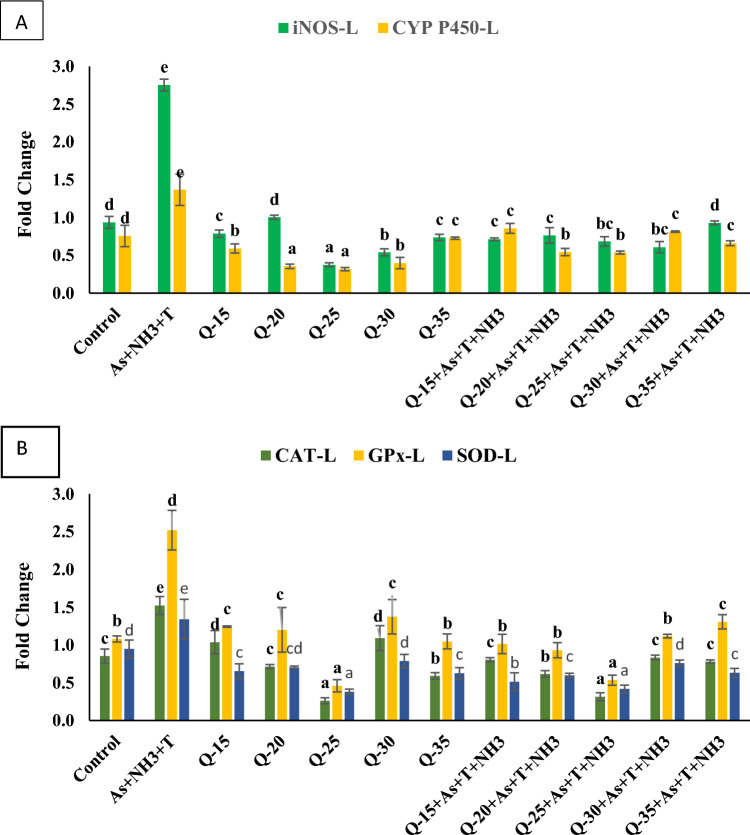
(**A**–**B**): Effect of quinoa husk on inducible nitric oxide synthase (*iNOS*), *CYP 450, CAT, GPx* and *SOD* in fish liver. Within endpoints and groups, bars with different superscripts differ significantly (**a**–**g**). Data expressed as Mean ± SE (n = 3).

In this study, we investigated the impact of replacing fishmeal with QH at levels of 15%, 20%, 25%, 30%, and 35% on *iNOS* and *CYP450* gene regulation in *P. hypophthalmus* over a period of 105 days. The fish were exposed to either a control condition or concurrent exposure to ammonia, arsenic, and high-temperature stress. The data are illustrated in Fig. 3A. Concurrent exposure to ammonia, arsenic, and high-temperature stress, along with a control diet, resulted in a noticeable upregulation of the gene expression of *iNOS* (*p* = 0.0016) and *CYP450* (*p* = 0.0021) in liver tissue compared to the control group and the QH-fed group. Intriguingly, the gene expressions of *iNOS* and *CYP450* were significantly downregulated in the groups fed with a 25% and 30% QH diet without stressors, followed by the groups fed with a 25% and 30% QH diet with stressors (As + NH_3_ + T), in comparison to the control group and the other experimental groups.

#### Catalase (*CAT*), Superoxide dismutase (*SOD*) and Glutathione peroxidase (*GPx*)

In the present investigation, we examined the gene expressions of *CAT, SOD*, and *GPx* in the liver tissue of *P. hypophthalmus* that were subjected to ammonia, arsenic, and high-temperature stress while being fed either a control diet or QH for 105 days. The data are summarized in Fig. 3B. The gene expressions of *CAT* (*p* = 0.001), *GPx* (p = 0.001), and *SOD* (*p* = 0.0013) in the liver were significantly upregulated in response to concurrent exposure to ammonia, arsenic, and high temperature (34 °C) compared to the control group and the QH-fed group. Surprisingly, the stress induced by ammonia, arsenic, and high temperature significantly downregulated *CAT, GPx*, and *SOD* in the liver tissue of *P. hypophthalmus* in the groups fed with a 25% QH diet, with and without stressors, compared to the control group and the other experimental groups. The groups fed with QH at 15%, 20%, 30%, and 35% were less effective in regulating the gene expressions of *CAT, GPx*, and *SOD*.

#### Gene involved in Immuno-modulation (*TNFα, IL, TLR* and *Ig*)

**Figure 4:**
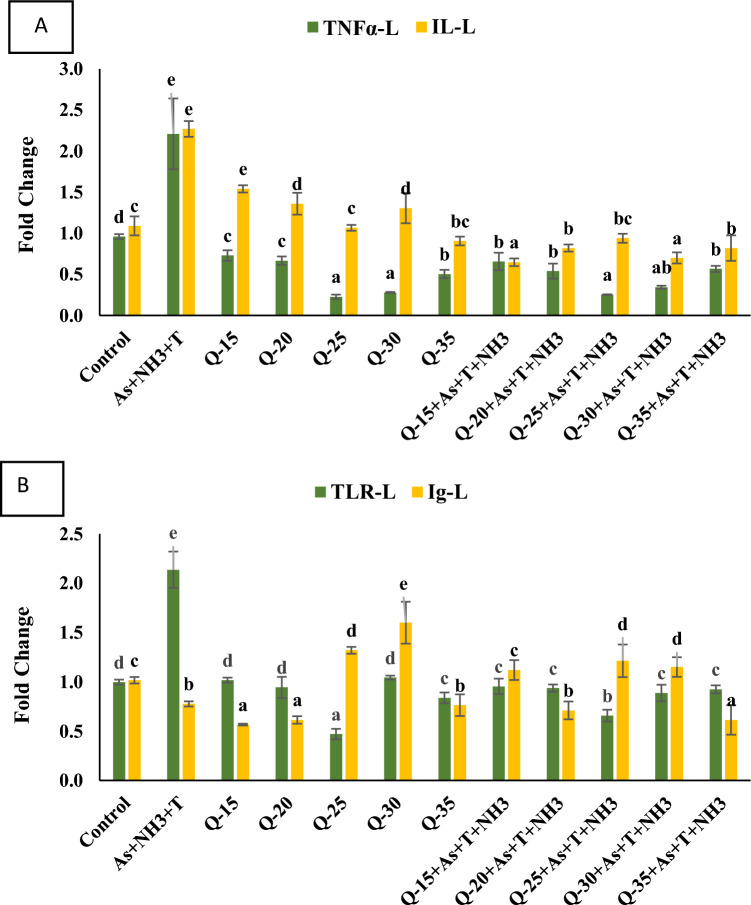
(**A**-**B**): Effect of quinoa husk on tumor necrosis factor (*TNF*), iterleukin (*IL*), toll like receptor (*TLR*) and immunoglobulin gene (*Ig*) regulation in fish liver. Within endpoints and groups, bars with different superscripts differ significantly (**a**–**g**). Data expressed as Mean ± SE (n = 3).

In this study, we assessed the immunological status of fish by examining the gene expression of *TNFα, IL, TLR*, and *Ig*. The data are presented in Fig. 4A, B. The gene expressions of *TNFα* (*p* = 0.0021) and *IL* (*p* = 0.0018) in liver tissue were significantly upregulated in response to concurrent exposure to ammonia, arsenic, and high-temperature stress (As + NH_3_ + T), compared to the control group and the QH-fed groups. Furthermore, the gene expression of *TNFα* was substantially downregulated in the group fed with QH at 25%, followed by the 30% group, with and without stressors, compared to the control group and the other QH-fed groups. Interestingly, the gene expression of *IL* was significantly downregulated in the groups fed with QH at 15% and 30% with stressors (As + NH_3_ + T), compared to the control group and the other experimental groups (Fig. 4A).

Similarly, the gene expression of *TLR* in the liver tissue of *P. hypophthalmus* was significantly upregulated (*p* = 0.0029) by As + NH_3_ + T, in contrast to the control group and the QH-fed groups. Conversely, the QH-fed group at 25%, without stressors, exhibited a noticeable downregulation compared to the control group and the other QH-fed groups. However, in contrast to the *TLR* results, the *Ig* gene expression was significantly downregulated (*p* = 0.0015) under As + NH_3_ + T conditions, while Ig was markedly upregulated in the groups fed with QH at 30% and 25%, with and without stressors, compared to the control group and the other experimental groups (Fig. 4B).

### Impact of quinoa husk (QH) on gene regulation involved tertiary stress response

#### Growth performance

The impact of different levels of QH at 15%, 20%, 25%, 30%, and 35% on growth performance, including final weight gain %, feed conversion rate (FCR), specific growth rate (SGR), protein efficiency ratio (PER), daily growth index (DGI), Thermal growth coefficient (TGC), and relative feed intake (RFI), was evaluated in *P. hypophthalmus*. The data for growth performance are presented in Table 4. Final weight gain % significantly improved (*p* = 0.0023) when QH was included at 25%, both with and without stressors (As + NH_3_ + T), compared to the control group and other QH feeding diets. Conversely, the lowest weight gain % was observed in the group concurrently exposed to arsenic, ammonia, and high-temperature stress and fed with the control diet. Similarly, SGR (*p* = 0.004), PER (*p* = 0.0031), DGI (*p* = 0.0018), and RFI (*p* = 0.0011) exhibited notable improvements with QH at 25%, with or without stressors, compared to the control group and other dietary treatments. Furthermore, the group treated with As + NH_3_ + T and fed the control diet displayed significantly the lowest SGR, PER, DGI, and RFI. Interestingly, the lowest FCR was notably observed in the groups fed with QH at 25% and 30%, both with and without stressors, compared to the control group and the other experimental groups.

**Table 4:** Effect of quinoa husk diets on growth performance (final body weight gain %, FCR, SGR, PER, DGI, TGC and RFI) of *P. hypophthalmus* reared under arsenic, ammonia and high temperature stress for 105 days.

Treatments	Final weight gain %	FCR	SGR	PER	DGI	RFI
Control	115.42^d^ ± 2.89	2.91^c^ ± 0.07	0.80^c^ ± 0.01	0.99^c^ ± 0.02	1.20^d^ ± 0.02	334.98^d^ ± 1.05
As + NH_3_ + T	67.53^a^ ± 3.72	4.20f. ± 0.22	0.50^a^ ± 0.0	0.69^a^ ± 0.03	0.77^a^ ± 0.03	281.93^a^ ± 1.66
Q-15	83.33^b^ ± 1.34	3.61^e^ ± 0.06	0.62^b^ ± 0.01	0.79^b^ ± 0.03	0.91^b^ ± 0.02	300.38^b^ ± 0.14
Q-20	84.08^b^ ± 1.94	3.57^e^ ± 0.07	0.62^b^ ± 0.02	0.82^b^ ± 0.04	0.93^b^ ± 0.01	299.98^b^ ± 1.11
Q-25	161.05^ g^ ± 2.31	2.25^a^ ± 0.03	0.89^d^ ± 0.01	1.39^ g^ ± 0.02	1.56f. ± 0.012	363.0f. ± 1.23
Q-30	139.21^e^ ± 2.76	2.45^b^ ± 0.06	0.89^d^ ± 0.03	1.27f. ± 0.03	1.39^e^ ± 0.01	340.39^e^ ± 1.61
Q-35	110.22^d^ ± 5.60	2.88^c^ ± 0.14	0.67^bc^ ± 0.02	1.08^de^ ± 0.07	1.17^d^ ± 0.02	315.66^ cd^ ± 1.22
Q-15 + As + T + NH_3_	100.69^c^ ± 1.45	3.10^c^ ± 0.05	0.66^bc^ ± 0.01	1.04^de^ ± 0.01	1.07^c^ ± 0.05	311.91^c^ ± 0.72
Q-20 + As + T + NH_3_	95.95^c^ ± 2.46	3.23^d^ ± 0.09	0.65^bc^ ± 0.01	0.89^bc^ ± 0.05	1.04^c^ ± 0.03	309.45^c^ ± 1.98
Q-25 + As + T + NH_3_	150.30f. ± 1.69	2.40^b^ ± 0.01	0.88^d^ ± 0.03	1.22^e^ ± 0.03	1.48f. ± 0.04	361.11f. ± 1.95
Q-30 + As + T + NH_3_	136.88^e^ ± 2.52	2.47^b^ ± 0.03	0.84^d^ ± 0.02	1.19^e^ ± 0.02	1.39^e^ ± 0.11	337.24^e^ ± 2.27
Q-35 + As + T + NH_3_	97.12^c^ ± 1.82	3.20^d^ ± 0.02	0.64^bc^ ± 0.01	0.99^c^ ± 0.04	1.05^c^ ± 0.13	310.50^c^ ± 2.83
P-value	0.0018	0.0011	0.0023	0.0024	0.0037	0.0055

Values in the same row with different superscript (a, b, c, d, e) differ significantly. Data expressed as Mean ± SE (n = 3). FCR: feed conversion ratio; SGR: specific growth rate; PER: protein efficiency ratio; DGI: Daily growth index; TGC: Thermal growth coefficient; RFI: relative feed intake.

#### Growth hormone gene regulation (*GH*)

**Figure 5:**
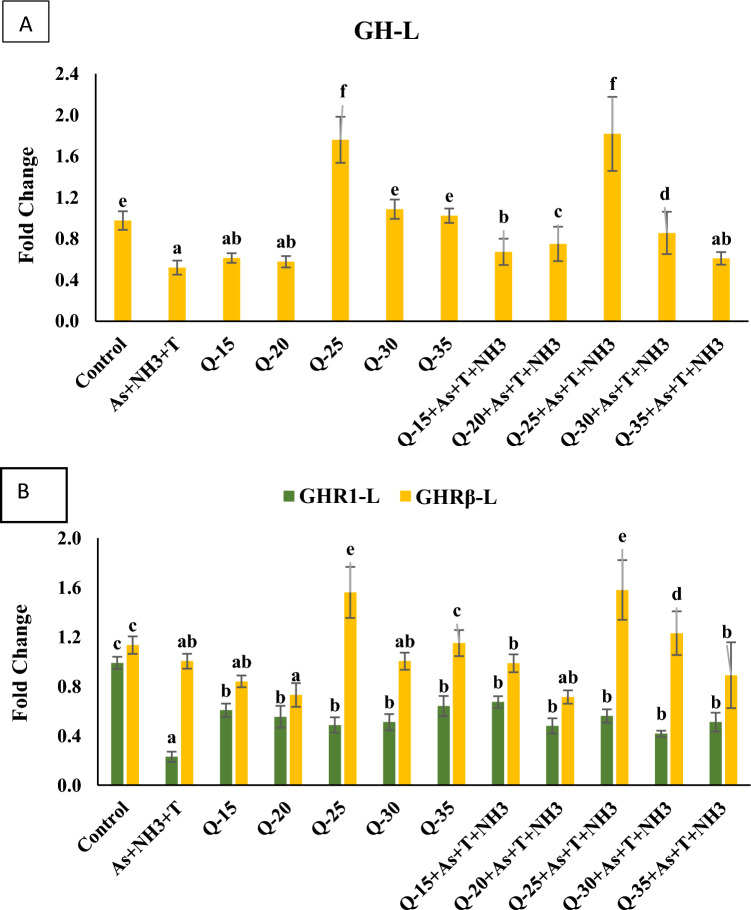
(**A**–**B**): Effect of quinoa husk on growth hormone (*GH*), growth hormoe regulator 1 and β (*GHR1* and *GHRβ*) in fish liver. Within endpoints and groups, bars with different superscripts differ significantly (**a**–**g**). Data expressed as Mean ± SE (n = 3).

The gene expression of *GH* was significantly downregulated (*p* = 0.0031) under concurrent exposure to ammonia, arsenic, and high-temperature stress in the groups fed with QH at 15% and 20%, compared to the control group and other treatment groups. Conversely, the group fed with QH at 25%, with and without stressors, exhibited substantial upregulation of *GH* gene regulation when compared to the control group and other experimental groups (Fig. 5A).

#### Growth hormone regulator (*GHR1 *and* GHRβ*)

The gene regulation of growth hormone regulator (*GHR1*) was significantly downregulated (*p* = 0.001) in response to concurrent exposure to ammonia, arsenic, and high-temperature stress (34 °C), compared to the control group and the QH-fed group. Conversely, *GHR1* was noticeably upregulated in the control diet group, followed by the QH-fed groups at 15%, 20%, 25%, 30%, and 35%, with and without stressors (As + NH_3_ + T). Similarly, *GHRβ* exhibited substantial downregulation in response to QH at 20%, followed by stressors (As + NH_3_ + T), compared to the control group. In contrast, *GHRβ* showed significant upregulation in the control diet group (Fig. 5B).

#### Insulin like growth factor (*IGF1X *and *IGF2X*)

**Figure 6:**
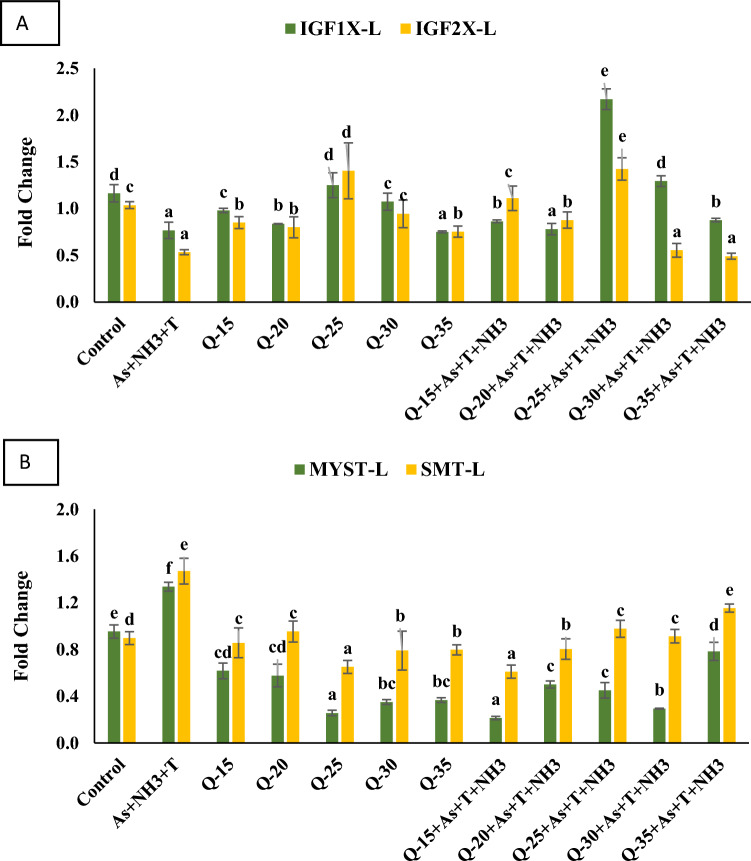
(**A**–**B**): Effect of quinoa husk on insuline like growth factor (*IGF1X* and *IGF2X*), myostatin (*MYST*) and somatostatin (*SMT*) gene regulation in fish liver. Within endpoints and groups, bars with different superscripts differ significantly (**a**–**g**). Data expressed as Mean ± SE (n = 3).

In this study, we examined the gene regulation of *IGF1X* and *IGF2X* in the liver tissue of *P. hypophthalmus* exposed to concurrent ammonia, arsenic, and high-temperature stress for 105 days. The data are presented in Fig. 6A. The concurrent exposure to arsenic, ammonia, and high-temperature stress significantly downregulated the gene expression of *IGF1X* (*p* = 0.0012) and *IGF2X* (*p* = 0.001) compared to the control group and the QH-fed groups. Conversely, the group fed with a QH diet at 25% with stressors (As + NH_3_ + T) exhibited a significant upregulation in the gene expression of *IGF1X* and *IGF2X* compared to the control group and the other experimental groups.

#### Myostatin and somatostatin (*MYST* and *SMT*)

The effects of different feeding groups with QH at 15%, 20%, 25%, 30%, and 35% on *P. hypophthalmus* were assessed under control conditions and concurrent exposure to ammonia, arsenic, and high-temperature stress for 105 days. The data for *MYST* and *SMT* gene expression are presented in Fig. 6B. Concurrent exposure to ammonia, arsenic, and high-temperature stress significantly upregulated the gene expressions of *MYST* (*p* = 0.001) and *SMT* (*p* = 0.001) compared to the control group and the various QH-fed groups. Interestingly, the gene regulation of *MYST* and *SMT* was downregulated in the group fed with QH at 25% without stressors and the group fed with QH at 15% with stressors, compared to the control group and the other experimental groups.

### Bioaccumulation of arsenic

The concentrations of arsenic in water and the bioaccumulation of arsenic in various fish tissues, including liver, gill, kidney, muscle, and brain, were determined in *P. hypophthalmus*. The data regarding arsenic bioaccumulation are presented in Table 5. In the experimental water, the arsenic concentrations in the groups exposed to As + NH_3_ + T and those fed with QH at 15%, 20%, 25%, 30%, and 35% with stressors were measured at 1850, 1547, 1658, 1441, 1724, and 1794 µg L^−1^, respectively. In contrast, the arsenic concentration in water was not detected in the groups fed with QH at 15%, 20%, 25%, 30%, and 35% without stressors. Furthermore, the highest bioaccumulation of arsenic was observed in the kidney and liver tissues, followed by the gill, muscle, and brain tissues.

**Table 5:** Effect of quinoa husk on arsenic concentration and bioaccumulation in water and different fish tissues of *P. hypophthalmus* reared under arsenic, ammonia and high temperature stress for 105 days.

Treatments	Water (µg L^-^1)	Liver (mg kg^−1^)	Gill (mg kg^−1^)	Kidney (mg kg^−1^)	Muscle (mg kg^−1^)	Brain (mg kg^−1^)
Control	BDL	BDL	BDL	BDL	BDL	BDL
As + NH_3_ + T	1850.46	5.12	4.36	5.96	1.12	0.97
Q-15	BDL	BDL	BDL	BDL	BDL	BDL
Q-20	BDL	BDL	BDL	BDL	BDL	BDL
Q-25	BDL	BDL	BDL	BDL	BDL	BDL
Q-30	BDL	BDL	BDL	BDL	BDL	BDL
Q-35	BDL	BDL	BDL	BDL	BDL	BDL
Q-15 + As + T + NH_3_	1547.28	4.18	3.85	5.18	0.89	0.47
Q-20 + As + T + NH_3_	1658.17	4.89	3.41	5.44	0.67	0.39
Q-25 + As + T + NH_3_	1441.16	4.96	3.62	4.01	0.45	0.41
Q-30 + As + T + NH_3_	1724.25	4.71	3.48	5.08	0.68	0.44
Q-35 + As + T + NH_3_	1794.63	5.19	3.59	5.32	0.89	0.49

Data expressed as Mean ± SE (n = 3). BDL: Below detection limit.

## Discussion

The present investigation focuses on replacing fishmeal with quinoa husk (QH) at varying levels (15%, 20%, 25%, 30%, and 35%) to assess its efficacy for overall fish development, including gene regulation. Additionally, the study explores the mitigating potential of QH against low doses of ammonia, arsenic, and high-temperature stress in *P. hypophthalmus*, classified into primary, secondary, and tertiary stress responses. The results indicate that simultaneous exposure to ammonia, arsenic, and high-temperature stress results in elevated cortisol levels. Generally, aquatic animals, including fish, require more energy to maintain body homeostasis during stressful conditions. Cortisol, in particular, facilitates glycogen decomposition in liver tissue to cope with stress^40^ and regulates blood glucose levels while controlling nutritional and physiological metabolism in fish^41^. Moreover, cortisol mobilizes amino acids, glucose, and free fatty acids to meet the immediate energy demands of the animal. However, excessive mobilization of these metabolites by cortisol can lead to a reduction in body and muscle mass due to increased energy expenditure^42^. Furthermore, arsenic can target multiple sites on the hypothalamus-pituitary-interrenal axis, potentially explaining the increased cortisol secretion and altered ACTH and cortisol levels^17,42^. Similarly, ammonia, being highly fat-soluble and capable of moving easily through the biofilm, can also affect cortisol levels^43^. The results also reveal that a QH diet at 25% significantly reduces cortisol levels. This suggests that the QH diet at 25% may actively stimulate the HPA axis and cell-mediated immune responses, resulting in lowered cortisol levels^44^. This study represents the first report on the role of quinoa husk (QH) in reducing cortisol levels in fish under multiple stress conditions. The induction of heat shock proteins (*HSP*) during stress is likely attributed to preventing protein aggregation and misfolding when exposed to simultaneous stressors such as As + NH_3_ + T, leading to the reorganization of protein homeostasis^45^. HSP proteins are also recognized for fortifying immunity during stressful conditions (Fu et al. 2011). In the case of ammonia toxicity, elevated NH_3_ levels in fish serum can decrease antioxidant levels and result in significant damage to HSP proteins^46^. Surprisingly, dietary supplementation of QH at 25% substantially downregulated the *HSP 70* gene. This effect of QH can be ascribed to its rich nutritional components and antioxidants, which safeguard cells against HSP protein denaturation. Concurrent exposure to As + NH_3_ + T induces the expression of DNA damage-inducible protein (DDIP) due to the production of reactive oxygen species (ROS) resulting from stress induced by As + NH_3_ + T. ROS production damages the respiratory chain of the mitochondrial membrane, modifies DNA bases, and disrupts the ribose ring structure, leading to DNA damage^47^. Exposure to As + NH_3_ + T also results in lipid, protein, and DNA damage, altering cellular function cascades and DNA methylation patterns^48^. Interestingly, the noticeable downregulation of the DDIP gene by QH at 25% could be attributed to its crucial roles in anti-cancer, antioxidant, and anti-hypertensive processes^49^.

In the current investigation, the gene regulation of *CYP 450, Cas 3a, 3b, MT, Na* + *K* + *ATPase*, and *iNOS* exhibited significant upregulation under simultaneous exposure to arsenic, ammonia, and high-temperature stress. The heightened expression of *CYP 450* could be attributed to alterations in metabolic pathways, such as the arachidonic acid, lipoxygenase, and cyclooxygenase pathways^50^. The metabolism of toxic substances in aquatic animals primarily involves oxidation, reduction, coupling reactions, and hydrolysis, with *CYP 450* playing a crucial role in activating these metabolic processes^51^. CYPs are pivotal in protecting cells against reactive oxygen species (ROS) and detoxifying toxic chemicals in living organisms, including fish^52^. *Caspase 3a* and *3b* are implicated in the apoptosis process, governing programmed cell death. These enzymes, recognized as aspartate-specific cysteine proteases during apoptosis pathways, regulate cell death receptors in mitochondria^53^. Dysfunctional mitochondria can release cytochrome c into the cytosol, activating caspase-3^54^. Exposure to As + NH_3_ + T stress upregulated the *MT* gene, possibly due to its binding with the C-terminal cysteine of MTF (metal regulatory transcription factor-1) and the high content of thiol groups^55^. Furthermore, iNOS was also upregulated with As + NH_3_ + T stress. Ammonia toxicity can regulate the L-amino acid transport system, leading to protein nitration and nitric oxide (NO) synthesis in tissues^56^. Higher NO generation in brain tissues may result from reactive oxygen and nitrogen species, enhancing protein nitration^57^. It has also been reported that NFκB, a ubiquitous transcription factor, inhibits *iNOS* and enhances NO generation in fish^58^. However, *iNOS* was substantially upregulated by stressors (As + NH_3_ + T). The *Na*^+^*K*^+^*ATPase* gene was significantly downregulated under concurrent exposure to As + NH_3_ + T. Interestingly, dietary QH at 25% notably improved the gene regulation of *CYP 450, Cas 3a, 3b, MT, Na* + *K* + *ATPase*, and *iNOS*. QH's role in enhancing gene regulation can be attributed to its properties of protein hydrolysis and various biological activities, including antioxidant, anti-diabetic, anti-cancer, anti-hypertensive, and anti-inflammatory activities^59–61^. QH has the potential to regulate and accelerate the Akt-signaling pathway and A375 cell apoptosis. Furthermore, QH has the potential to regulate apoptosis and detoxification via *Cas 3* and *3b*, as well as *CYP 450*^62^. This study is the first to report on the role of QH in the gene regulation of *CYP 450, Cas 3a, 3b, MT, Na* + *K* + *ATPase*, and *iNOS* for mitigating multiple stresses (As + NH_3_ + T) in *P. hypophthalmus*.

Furthermore, anti-oxidative genes such as *CAT, GPx,* and *GST* were upregulated by As + NH_3_ + T. The anti-oxidative status was significantly improved with a QH diet at 25% inclusion. Interestingly, *SOD, GPx*, and *CAT* are essential free radical scavenging enzymes that efficiently regulate free radicals^63,64^. Han et al^49^. reported that QH could regulate *SOD* gene expression to maintain the fish's anti-oxidative status. The present investigation reveals that QH has an excellent anti-oxidative effect, controlling the gene regulation of *SOD, CAT*, and *GPx* to mitigate multiple stresses (As + NH_3_ + T). Immunological attributes, such as the gene regulation of *TNFα, IL, TLR*, and *Ig*, were altered under concurrent exposure to arsenic, ammonia, and high temperature. However, gene regulation of immunological attributes notably improved with a QH diet. The results demonstrate the role of QH in enhancing fish immunity by regulating the gene expression of *TNFα, IL, TLR*, and *Ig*. Quinoa regulates the immune response by activating signaling pathways and upregulating the expression of cytokine genes such as *TNFα, IL-6, TLR*, and *Ig*, resulting in improved fish immunity^65,66^.

Growth performance indicators, such as weight gain %, SGR, FCR, PER, DGI, and RFI in *P. hypophthalmus*, were significantly improved by a dietary QH at 25%. Moreover, concurrent exposure to As + NH_3_ + T significantly reduced growth performance. Arsenic, ammonia, and high-temperature stress downregulated genes responsible for growth performance, including *GH, GHR1, GHRβ, IGF1X, IGF2X*, while upregulating the *MYST* and *SMT* gene regulation. Reduced growth can be attributed to decreased feed intake, a lower specific growth rate, immune suppression, and tissue erosion in fish^67–70^. The study conducted by Yu et al^69,70^. demonstrated that the fish fed with *Taraxacum mongolicum* polysaccharide enhances the growth performance. Elevated temperatures can accelerate bioavailability, chemical reactions, and diffusion rates, further impacting growth^71^. Ammonia and arsenic are known to be highly toxic, leading to growth retardation and mass mortality in fish^72^. The growth performance is also related to the bioaccumulation of arsenic, which cannot be efficiently metabolized and can induce toxicity and growth reduction in fish^73^. Interestingly, dietary QH assists in the binding of the *GH* gene with *GHR* to regulate the growth and development of fish, thereby influencing *SMT*, dopamine, ghrelin, and GnRH^74^. However, *MYST* suppresses myoblasts and reduces growth, likely due to terminal differentiation and fiber enlargement, regulated by glucocorticoids^75^. Notably, *MYST* negatively regulates muscle growth through the activation of the Mstn/Smad pathway, inhibiting the transcription of myogenic factors that regulate muscle cell differentiation and proliferation. It also hinders muscle growth by blocking the Akt/mTOR pathway, reducing protein synthesis^76^. The results regarding arsenic bioaccumulation in water and different fish tissues indicate that dietary QH may not significantly affect the detoxification process of arsenic. However, compared to the As + NH_3_ + T treatment, groups fed with QH showed reduced bioaccumulation of arsenic in fish tissues.

## Conclusion

The current investigation focuses on substituting fishmeal with quinoa husk (QH) at varying levels: 15%, 20%, 25%, 30%, and 35%. The study evaluates QH's effectiveness in mitigating stress gene responses to concurrent exposure to arsenic, ammonia, and high temperatures in *P. hypophthalmus*. This research assesses QH's impact on gene regulation related to growth performance, immunity, antioxidative status, apoptosis, genotoxicity, and detoxifying genes in *P. hypophthalmus*. Notably, QH at a 25% inclusion rate demonstrates a significant capacity to mitigate the stress response induced by As + NH_3_ + T through the regulation of genes involved in these processes. Furthermore, replacing 25% of fishmeal with QH appears to yield favourable outcomes in enhancing growth performance and modulating immune responses against multiple stressors in fish.

## Data Availability

The datasets generated during and/or analyzed during the current study are available from the corresponding author on reasonable request.

## Abbreviations

QH: Quinoa husk
As: Arsenic
NH_3_: Ammonia
T: High temperature
*NFkB*: Nuclear factor kappa B
*iNOS*: Inducible nitric oxide synthase
*HSP70*: Heat shock protein
*MT*: Metallothionine
*CAT*: Catalase
*SOD*: Superoxide dismutase
*GPx*: Glutathione peroxidase
*CAS 3a*: Caspase 3a
*TNFα*: Tumor necrosis factor
*IL*: Interleukin
*TLR*: Toll-like receptors
*GH*: Growth hormone
*GHR1; GHRβ*: Growth hormone regulator 1 and β
*MYST*: Myostatin
*SMT*: Somatostatin
IPCC: Intergovernmental panel on climate change
TAN: Total ammonia nitrogen
RAC: Research advisory committee
PME: Prioritization, monitoring and evaluation
LC_50_: Lethal concentration
CMC: Carboxymethyl cellulose
CP: Crude protein
EE: Ether extract
FEC: Feed conversion efficiency
SGR: Specific growth rate
PER: Protein efficiency ratio
DGI: Daily growth index
TGC: Thermal growth coefficient
RFI: Relative feed intake
ICPMS: Inductively coupled plasma mass spectrometry
GnRH: Gonadotropin releasing hormone
